# Time to negative PCR in various disease categories of COVID-19 infection in Pakistani population

**DOI:** 10.12669/pjms.38.1.4476

**Published:** 2022

**Authors:** Kaleem Ullah Toori, Asma Chaudhry, Muhammad Arsalan Qureshi

**Affiliations:** 1 Dr. Kaleem Ullah Toori, FRCP (Glasgow) Department of Medicine, KRL Hospital, Islamabad, Pakistan; 2 Dr. Asma Chaudhry, MRCP (UK), FCPS General Medicine, (Pakistan) Department of Medicine and Endocrinology, Southend University Hospital, Southend-on-Sea, United Kingdom; 3 Dr. Muhammad Arsalan Qureshi (MBBS) Department of Medicine, KRL Hospital, Islamabad, Pakistan

**Keywords:** Corona Virus Induced Disease (COVID-19), SARS-CoV-2, age, gender, pre-morbidities, disease severity, time to negative PCR

## Abstract

**Objectives:**

To identify association of epidemiological characteristics, presence of underlying pre-morbidities and disease severity with time to first negative PCR in Corona virus disease 2019.

**Methods:**

Total 842 Corona Virus Real Time Polymerase-Chain-Reaction positive patients were included in this cross-sectional study. Patients were admitted to Department of Medicine at KRL Hospital Islamabad from April to August 2020. Age, gender, symptoms, pre-morbidities and disease severity were recorded. Outcome (recovered versus died) was documented. World Health Organization categories to classify disease severity (asymptomatic, mild, moderate and severe) were used. Time to negative PCR was documented as time between first positive PCR to first negative PCR.

**Results:**

The mean age of patients was 39.04 ± 11.32 years with 99.8 % being males. Majority of patients (78.4%) were asymptomatic. Amongst symptoms, fever was the most common symptom. Diabetes mellitus and hypertension were the most commonly recorded co-morbidity. Mean time to negative PCR was 8.8 ± 3.1 days. A large proportion of patients recovered (99.9%). Significant positive correlation (p value < 0.05) was found between age, gender, presence of underlying pre-morbidities and disease severity categories with time to first negative PCR.

**Conclusion:**

The underlying epidemiological factors, pre-morbidities and disease severity are associated with time to negative PCR and hence affect frequency of recovery samples.

## INTRODUCTION

A novel human coronavirus, severe acute respiratory syndrome coronavirus 2 (SARS-CoV-2), has alarmed the world with rapid spread across the globe. It was first identified in Wuhan, China, in December 2019 and quickly declared a pandemic in March 2020 by World Health Organization (WHO).^1^ SARS-CoV-2 has infected humans randomly regardless of age, ethnicities, gender or comorbidities, however gradually patterns have been noted as to which groups are more susceptible to this lethal virus. SARS-CoV-2 has a wide spectrum of clinical presentation involving multiple systems at time. The severity of the disease can range from being completely asymptomatic to ending up in septic shock and acute respiratory distress syndrome (ARDS) requiring mechanical ventilation. To make things more complicated, currently there are reports out of the United Kingdom regarding a mutated strain with even quicker spread.^2^

Much remains to be learned, however it has been established that virological detection of SARS-CoV-2 through Reverse transcription polymerase chain reaction (RT-PCR) is the gold standard for diagnosing infection.^3^ Understanding virus detectability during the disease process is vital, firstly to establish patterns, and secondly to understand the duration of infectivity in individuals based on virus positivity. It is necessary to know the viral shedding patterns and how disease severity affects it in order to ensure optimum epidemiological control strategies to further halt the virus spread and control the disease burden. Multiple studies have been conducted worldwide that have tried to describe how disease severity and other factors influence viral shedding in patients affected and propose mean duration of viral shedding.^4^-^6^

As new literature comes to light, management of the disease is being evolved in a similar fashion. We aimed to see if some epidemiological factors and disease severity affect the time required for the conversion of RT-PCR from positive to negative. To the best of our knowledge ample research has not taken place locally and, with our efforts we intend to add to the literature, which would help determine the right time to take recovery RT-PCR samples in a Pakistani population to decrease economic burden on a resource constraint system.

## METHODS

We did a prospective cross sectional study at KRL Hospital Islamabad. The study duration was of three months from 1^st^ April to 31^st^ August 2020. Sample size calculation was not applicable as there is no data on prevalence of the disease in Pakistani population or internationally.^7^ All patients who presented to us with symptoms of SARS-CoV-2 were first isolated in the designated facility for COVID-19 patients at KRL Hospital. Nasopharyngeal swabs were taken as per WHO protocol.^8^ Sample also included asymptomatic patients who were randomly screened and found incidentally to have a positive result. All consecutive patients with a positive RT-PCR were included in the study. Informed consent was taken from the patients and the study was approved by the ethical board review of the hospital (Ref ERC: KRL-HI-ERC/Dec20/28; dated March 31, 2021).

Complete history and demographic profile were obtained. A diagnosis of COVID-19 was made based on typical clinical manifestations and positive RT-PCR for SARS-CoV-2. All necessary investigations and imaging was done as per the hospital’s protocol. Case severity definitions were as per the interim guidance of WHO.^9^ Asymptomatic (patients are RT-PCR positive but do not show symptoms), mild (patients are RT-PCR positive but no hypoxia), moderate (RT-PCR positive patients who show signs of pneumonia and but no signs of severe hypoxia with Spo2 > 90%), severe (signs of severe pneumonia evident with respiratory rate more than 30 breaths/minute or Spo2 < 90% and critical (with Acute Respiratory Distress Syndrome [ARDS] or septic shock). Both severe and critical patients were included under the category of severe disease.

Time of the disease onset was taken as the first date of beginning of signs and symptoms of COVID-19 or the date of the first positive PCR in asymptomatic patients.^10^ The time to negative PCR was defined as the duration from the disease onset or PCR positivity to the first negative PCR, which was done on Day 5, 7, 11, 14 and then every 5^th^ day subsequently as needed. Discharge criteria of patients consisted of absence of fever and at least two consecutive negative RT-PCRs done ≥24 hours apart. Outcome was defined as recovered patients (PCR becoming negative) or patients who died.

Statistical analysis was performed using IBM SPSS 22. Continuous variables like age and time to negative PCR were expressed as mean values with standard deviation. Frequency and percentages were calculated for categorical variables like cases as per severity, symptoms, comorbidities and outcome. Pearson’s correlation was used to correlate parameters (age, gender, disease severity and presence of comorbidities) with time to negative PCR. Mean values among categorical groups were calculated and compared for statistical significance using ANOVA. P-value less than 0.05 was considered statistically significant. Confidence interval was at 95%.

## RESULTS

A total of 842 patients were included in this study; time to first negative PCR was recorded for these patients. The baseline demographics, frequency of symptom severity, pre-morbidity and outcome frequencies are presented in Table-I. The mean age was 39.04 ± 11.32 years. Male gender was predominant. Majority patients were asymptomatic i.e. 78.4 % (n=660). Fever and dry cough were the most common symptoms present amongst the symptomatic patients. Underlying pre-morbidities were present only in 12.8% (n=108) patients. Mean time to negative PCR was 8.8 ± 3.1 days. A large proportion of patients recovered i.e. 99.9% (n=841) of patients recovered with a very low mortality rate of 0.1% (n=1).

**Table-I:** Demographics and frequencies (n=842).

Characteristics	COVID-19 patients
Age (in years) – Mean ± SD	39.04 ± 11.3
***Gender- n (%)***	
Male	840 (99.8%)
Female	2 (0.2%)
***Disease Severity- n (%)***	
Asymptomatic	660 (78.4%)
Mild	144 (17.1%)
Moderate	21 (2.5%)
Severe	17 (2%)
***Symptoms - n (%)***	
Fever	158 (18.8 %)
Dry Cough	100 (11.9 %)
Shortness of breath	39 (4.6%)
Myalgia	53 (6.3%)
Sore throat	34 (4.0%)
Diarrhea	7 (0.8%)
Headache	7 (0.8%)
Rhinorrhoea	5 (0.6%)
Haemoptysis	1 (0.1%)
***Pre-morbidity present - n (%)***	n=108 (12.8%)
***Individual Pre-morbidities - n (%)***	
Diabetes Mellitus	57 (6.8%)
Hypertension	54 (6.4%)
Ischemic Heart Disease	22 (2.6%)
Chronic Respiratory Disease	14 (1.7%)
Chronic Kidney Disease	9 (1.1%)
Chronic Liver Disease	2 (0.2%)
Chronic Neuro-psychiatric condition	1 (0.7%)
Malignancy	1 (0.1%)
Time to First negative PCR (in days) – Mean ± SD	8.8 ± 3.1
***Outcome- n (%)***	
Recovered	841 (99.9 %)
Died	1 (0.1 %)

PCR-Polymerase Chain Reaction.

We analysed whether the time to first negative PCR was linked to age, gender, disease severity categories and pre-morbidities. As seen in results in Table-II, increasing age, male gender, increased disease severity and presence of pre-morbidities led to greater time to first negative PCR; these were significant results.

**Table-II:** Correlation of parameters with Time to First Negative PCR-Pearson’s Correction Analysis.

Characteristics	Time to Negative PCR r (p)
Age	0.11 (0.00) ^**^
Gender	0.08 (0.01) ^*^
Disease Severity	0.27 (0.00) ^**^
Premorbidties	0.17 (0.00) ^**^

p- Correlation is significant at the 0.05 level (2-tailed).

r- Pearson’s correlation coefficient.

We also evaluated whether there was a significant association between time to first negative PCR across all four categories of disease severity as reported in the Table-III. It can be seen that as the disease severity increases, the mean time to negative PCR increases steadily as well. This was significant as analyzed by ANOVA suggesting that greater disease severity leads to significantly prolonged time to first negative PCR.

**Table-III:** Time to First negative PCR amongst Disease Severity categories- ANOVA analysis.

Disease Severity Categories	N	Mean	Standard Deviation	Minimum	Maximum	ANOVA F Statistic (d.o.f), p- value
Asymptomatic	660	8.54	2.83	5	34	*33.30* (3,838), 0.00
Mild	144	8.84	3.11	5	23	
Moderate	21	13.28	4.27	8	21	
Severe	17	13.64	3.62	9	20	
Total	842	8.81	3.10	5	34	

d.o.f - degree of freedom.

## DISCUSSION

In our study, we reviewed some factors that are associated with time required for PCR to become negative in patients affected with COVID-19. As of yet there is incomplete understanding of the factors which influence the viral PCR negativity time in the local population. Our study is an attempt to recognize these factors at a tertiary care facility. It has been understood through previous studies and clinical experience that COVID-19 not only has a wide range of presentations and duration but a varying immune response as well.^11^ Whether prolonged PCR positivity is correlated with infectivity is still unclear^12^, but there are viral culture studies which suggest that the viable virus may not be present after an average of 8 days of symptom onset.^13^,^14^ Our findings are in line in with the above-mentioned mean number of days.

It has been postulated that SARS-COV-2 uses the Angiotensin Converting Enzyme 2^15^ to affect the host cells and gender based variations in their expression may lead to increased severity in males as compared to females. Our findings also relate with this hypothesis that males are more prone to be affected by COVID-19. We also noted that male gender was significantly associated with prolonged viral shedding which is seconded by a Chinese study.^16^

Agreeing with multiple studies^6^,^16^,^17^ our study also established increasing age as a risk factor for prolonged viral shedding which may be attributed to immunosenescence with advancing age leading to more susceptibility to infection and presence of underlying comorbid conditions which may lead to disease progression.

Although majority of the patients affected by SARS-COV -2 have mild forms of disease, a small number of patients acquire severe disease with possible life-threatening consequences. It is noteworthy that disease severity is also associated with time required for PCR negativity. Increasing severity is related with worsening hypoxia, ARDS and the requirement of mechanical ventilation. Since SARS –COV 2 affects the lower respiratory tract, it is likely for viral PCR to remain positive for longer as in these patients samples are taken from endotracheal aspirates. 16 This relationship with disease severity and PCR conversion time is not exclusive to COVID-19; during the MERS-COV (Middle East respiratory syndrome coronavirus infection) outbreak back in 2012, it was also noted that disease severity and detection of sub genomic mRNA were associated with each other.^18^

We also found that presence of underlying disease comorbidities is also associated with increased PCR positivity time. Diabetes and hypertension were most prevalent premorbid conditions in our study population. Our results are in line with Chinese studies which concluded that presence of any underlying disease was a risk factor for having more severe disease and prolonged viral shedding as compared to those without any premorbid conditions.^6^,^19^

### Limitations of the Study:

It included predominantly male patients as being organization employees, all males were to be admitted if they tested positive. On the other hand, females opted to isolate at home and were only admitted if they had moderate to severe disease requiring hospital management. Secondly, this was a single-centred study. Thirdly we had to exclude patients who did not have time to negative PCR documented for certain reasons which reduced our data cohort.

## CONCLUSION

We have identified age, gender, presence of premorbid conditions and disease severity to prolong the time for RT-PCR to become negative. This can potentially lead to avoid early recovery sampling and hence save burden of both money and time.

### Authors Contribution:

**KT:** Conceived, designed and did statistical analysis along with review of manuscript, he is accountable for the accuracy of study.

**MAQ:** Contributed to data collection and manuscript writing.

**AC:** Did manuscript writing with final review of manuscript.

## References

[1] Cucinotta D, Vanelli M (2020). WHO declares COVID-19 a pandemic. Acta Biomed.

[2] British Broadcasting Company (2020). New coronavirus variant:What do we know?.

[3] Centers for Disease Control and Prevention (2020). CDC diagnostic tests for COVID-19 August.

[4] Liu Y, Yan LM, Wan L, Xiang TX, Le A, Liu JM (2020). Viral dynamics in mild and severe cases of COVID-19. Lancet Infect Dis.

[5] Xiao AT, Tong YX, Gao C, Zhu L, Zhang YJ, Zhang S (2020). Dynamic profile of RT-PCR findings from 301 COVID-19 patients in Wuhan, China:a descriptive study. J Clin Virol.

[6] Feng Z, Li J, Yao S, Yu Q, Zhou W, Mao X (2020). Clinical factors associated with progression and prolonged viral shedding in COVID-19 patients:a multicenter study. Aging Dis.

[7] Guan WJ, Liang WH, Zhao Y, Liang HR, Chen ZS, Li YM (2020). Comorbidity and its impact on 1590 patients with COVID-19 in China:a nationwide analysis. Eur Respir J.

[8] World Health Organization (2020). Laboratory Testing for 2019 novel coronavirus (2019-ncov) in suspected human cases March.

[9] World Health Organization (2020). Clinical management of COVID-19:interim guidance May 2020 [Internet] Who.int.com.

[10] Fu Y, Li Y, Guo E, He L, Liu J, Yang B (2020). Dynamics and Correlation Among Viral Positivity, Seroconversion, and Disease Severity in COVID-19:A Retrospective Study. Ann Intern Med.

[11] Mathew D, Giles JR, Baxter AE, Oldridge DA, Greenplate AR, Wu JE (2020). Deep immune profiling of COVID-19 patients reveals distinct immunotypes with therapeutic implications. Science.

[12] Widders A, Broom A, Broom J (2020). SARS-CoV-2:The viral shedding vs infectivity dilemma. Infect Dis Health.

[13] Bullard J, Dust K, Funk D, Strong JE, Alexander D, Garnett L (2020). Predicting infectious SARS-CoV-2 from diagnostic samples. Clinical Infect Dis.

[14] Wolfel R, Corman VM, Guggemos W, Seilmaier M, Zange S, Müller MA (2020). Virological assessment of hospitalized patients with COVID-2019. Nature.

[15] Zhang H, Penninger JM, Li Y, Zhong N, Slutsky AS (2020). Angiotensin-converting enzyme 2 (ACE2) as a SARS-CoV-2 receptor:molecular mechanisms and potential therapeutic target. Intensive Care Med.

[16] Xu K, Chen Y, Yuan J, Yi P, Ding C, Wu W (2020). Factors associated with prolonged viral RNA shedding in patients with Corona virus disease 2019. Clin Infect Dis.

[17] Chen X, Zhu B, Hong W, Zeng J, He X, Chen J (2020). Associations of clinical characteristics and treatment regimens with the duration of viral RNA shedding in patients with COVID-19. Int J Infect Dis.

[18] Park WB, Poon LL, Choi SJ, Choe PG, Song KH, Bang JH (2018). Replicative virus shedding in the respiratory tract of patients with Middle East respiratory syndrome coronavirus infection. Int J Infect Dis.

[19] Guan WJ, Ni ZY, Hu Y, Liang WH, Ou CQ, He JX (2020). Clinical characteristics of coronavirus disease 2019 in China. N Engl J Med.

